# Effect of Diethylstilbestrol on Implantation and Decidualization in Mice

**DOI:** 10.3390/ijms26094122

**Published:** 2025-04-26

**Authors:** Feng Ran, Si-Ting Chen, Meng-Yuan Li, Dan-Dan Jin, Zeng-Ming Yang

**Affiliations:** 1 College of Animal Science, Guizhou University, Guiyang 550025, China; 2 College of Veterinary Medicine, South China Agricultural University, Guangzhou 510642, China

**Keywords:** diethylstilbestrol, implantation, uterine receptivity, pyroptosis

## Abstract

Diethylstilbestrol (DES) is a synthetic non-steroidal estrogen, which was widely used to prevent preterm birth and abortion from the 1940s to the 1970s. DES can increase the incidence of infertility, the abnormal reproductive tract, and autoimmune diseases. However, the mechanism underlying DES on early pregnancy in mice is unclear. This study evaluated the effects of DES on early pregnancy in mice, especially on uterine receptivity and decidualization. Newborn female mice were subcutaneously injected with 0.1 mg/kg DES, 1 mg/kg DES, or sesame oil as controls for 5 consecutive days. At 6 weeks old, these female mice were mated with 8–12-week-old fertile males to obtain pregnancy. The uteri of these mice were collected on days 4, 5, and 8 of pregnancy for further analysis. On days 5 and 8 of pregnancy, the number of implantation sites in 0.1 mg/kg DES group is similar to the control group, while almost no implantation sites are detected in the 1 mg/kg DES group. On day 4 of pregnancy, there was no significant difference in uterine receptive molecules between the control group and the 0.1 mg/kg DES group. However, the levels of uterine receptive molecules in the 1 mg/kg DES group are abnormal. In addition, 6 μM DES significantly inhibits mouse in vitro decidualization. The excessive activation of pyroptosis may lead to pregnancy failure. The pyroptosis-related molecules in the 1 mg/kg DES group were significantly up-regulated, suggesting that DES may contribute to pregnancy failure by over-activating pyroptosis.

## 1. Introduction

Diethylstilbestrol (DES) is an endocrine-disrupting chemical first produced by Dodds and colleagues in London, England, in 1938 [1,2]. The function of DES is similar to that of natural estradiol, and its potency is five times that of estradiol [3,4]. From the late 1940s to the 1970s, DES was widely used in pregnant women to prevent miscarriage and other complications [5]. However, women exposed to DES have an increased risk of breast cancer, and can cause vaginal clear cell adenocarcinoma of their daughters and testicular cancer of their sons [6,7]. When neonatal mice are exposed to 2 μg DES, 90% of the mice develop uterine adenocarcinoma at 18 months of age [8]. DES is prohibited from use in human pharmacy and clinical treatment due to its carcinogenic effects [9]. Because DES can promote protein synthesis and metabolism, livestock production still has illegal use [10]. DES residues are present in fish, milk, and the meat of some livestock in many countries [11]. Monitoring of environmental estrogens in sewage and surface water from different Asian locations found that DES concentrations are about 0.1–10 ng/L [9]. Compared with natural estrogen, DES is more stable and has a longer residence time in the body [12]. When a single oral dose of 10 mg DES was given to cattle, its half-life in cattle was 17 h [13]. When steers were exposed to DES by a single oral administration, DES residues could be detected in the small intestine, feces, and urine after 10 days [14]. DES can bioaccumulate in the human body via trophic transfer within the food chain, thereby perturbing the synthesis and release of physiological secretions [15].

DES exposure generally has adverse effects on reproduction. In humans, exposure to DES during pregnancy increases the incidence of genital tract cancer and leads to early menopause in their daughters [16]. In mice, 40 μM DES interferes with mouse oocyte maturation and reduces the first polar body extrusion (PBE) in vitro [11]. Feeding mice with 0.02, 0.2, and 2.0 ng/g of DES between days 11 and 17 of pregnancy increases the prostate weight in male offspring [17]. When rats are exposed to DES from 14.5 to 16.5 of pregnancy, the function of fetal Leydig cells and the expression of fetal androgen are affected [18]. Many DES-related structural and cellular abnormalities are caused by changes in gene programming, such as Hox and Wnt, which play an important role in genital tract differentiation [19]. DES can also affect the expression of estrogen receptor α, the progesterone receptor, and homeobox A11 related to uterine implantation in neonatal rats, and reduce the number of implantation sites [20]. However, the specific mechanism regarding how DES affects embryo implantation and decidualization is unclear.

Embryo implantation involves an interaction between a receptive uterus and a competent blastocyst [21]. Implantation and decidualization are a proinflammatory state, and a moderate proinflammatory response is conducive to blastocyst implantation [22,23]. Endometrial biopsy can lead to local damage and inflammation, but can increase uterine receptivity [24]. Decidual formation in pseudopregnant rodents may be caused by local damage such as suture of the uterine horn and intrauterine injection of oil [25]. DES exposure can cause prostate inflammation in rats and activate inflammatory pathways in mouse myometrium stem cells [26,27]. Pyroptosis is a lytic and inflammatory programmed cell death pathway initiated by inflammasomes and carried out by pore-forming gasdermin proteins [28]. It is characterized by membrane pore formation, swelling, and subsequent cytolysis combined with the release of pro-inflammatory intracellular contents, including interleukin (IL)-1β and IL-18 [29]. Excessive pyroptosis may result in persistent inflammatory responses, thereby contributing to the onset of inflammatory diseases [28]. In typical pyroptosis, the stimulation of bacteria and viruses is recognized by pattern recognition receptors, thereby activating the oligomerization of Nod-like receptor (NLR) family hot protein domain 3 (NLRP3) [30]. The oligomerized NLRP3 aggregates contain CARD (ASC) apoptosis-related spot-like proteins and recruit CASPASE 1 to form the NLRP3-ASC-CASPASE 1 protein complex [31]. CASPASE 1 can cleave GSDMD into N-terminal GSDMD (N-GSDMD) for forming holes on the cell membrane through releasing mature inflammatory factors IL-1β and IL-18 to promote the inflammatory response [32,33]. In the non-classical pathway, LPS activates CASPASE 4/5 or mouse CASPASE 11, leading to Caspase oligomerization and cleavage of GSDMD [34,35].

In this study, we subcutaneously injected 0.1 mg/kg DES and 1 mg/kg DES into newborn female mice for 5 consecutive days and mated at 6 weeks old with fertile males. Our results showed that exposure to DES impairs implantation and decidualization in mice and leads to abnormal uterine receptivity on day 4 of pregnancy. Meanwhile, we found that DES can over-activate pyroptosis, which may be the cause of pregnancy failure.

## 2. Results

### 2.1. Effects of DES on Implantation and Decidualization

**Figure 1:**
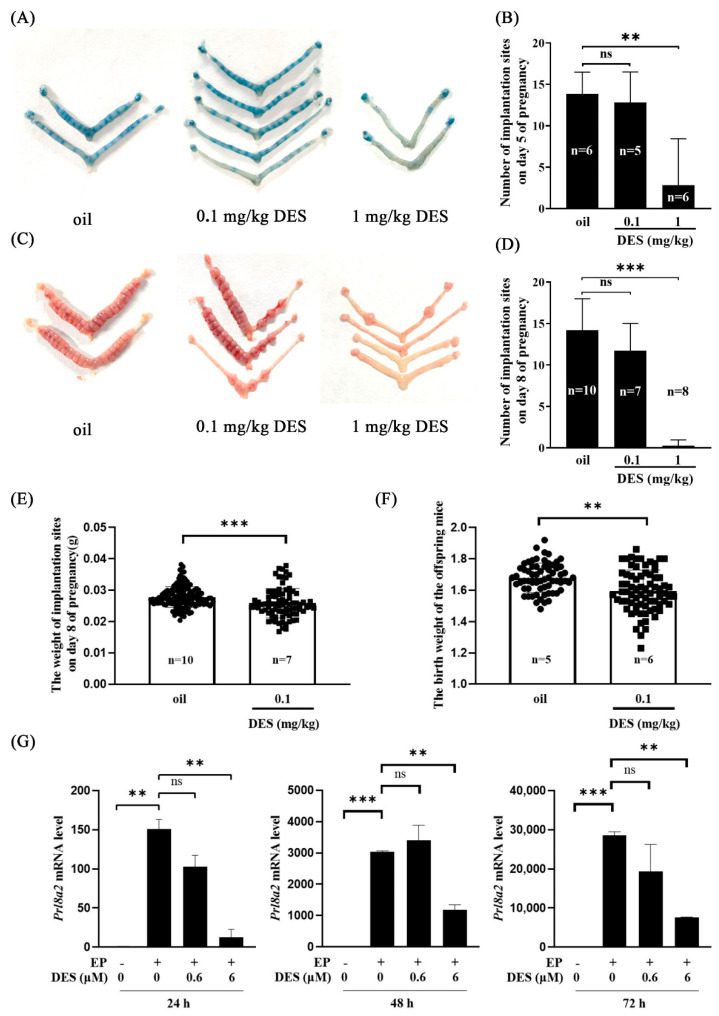
Effects of DES on implantation and decidualization in mice. (**A**) Morphology of the implantation site on day 5 of pregnancy in the control and DES treatment groups (0.1 mg/kg and 1 mg/kg). (**B**) The number of implantation sites on day 5 of pregnancy in the control and the DES treatment groups (0.1 mg/kg and 1 mg/kg). (**C**) Morphology of the implantation site on day 8 of pregnancy in the control and DES treatment groups (0.1 mg/kg and 1 mg/kg). (**D**) The number of implantation sites on day 8 of pregnancy in the control and DES treatment groups (0.1 mg/kg and 1 mg/kg). (**E**) The weight of implantation site on day 8 of pregnancy in the control and the DES-treated group (0.1 mg/kg). (**F**) The birth weight of offspring mice in the control and 0.1 mg/kg group. *n*, the number of mice used. (**G**) QPCR analysis on *Prl8a2* mRNA levels in mouse stromal cells treated with 0.6 μM and 6 μM DES for 24, 48, and 72 h after decidualization. EP, estradiol-17β and progesterone. **, *p* < 0.01; ***, *p* < 0.001; ns, not significant.

To explore the effect of DES on early pregnancy, we examined mouse uteri in each group on days 5 and 8 of pregnancy. Compared with the control group and 0.1 mg/kg DES group, the number of implantation sites in the 1 mg/kg DES group was significantly reduced on days 5 and 8 of pregnancy (Figure 1A–D). Compared with the control group, the weight of the implantation site on day 8 of pregnancy and the birth weight of their offspring were significantly reduced in the 0.1 mg/kg DES group (Figure 1E,F).

Decidualization is essential for pregnancy in mice. The prolactin family *Prl8a2* is a reliable marker of decidualization in vitro [36]. To further explore the effect of DES on decidualization in mice, mouse stromal cells were treated with two different concentrations of DES (0.6 μM and 6 μM) for 24 h, 48 h, and 72 h under in vitro decidualization. The mRNA level of *Prl8a2* was significantly decreased by 6 μM DES, while 0.6 μM DES did not affect the expression of *Prl8a2* (Figure 1G). These results indicate that DES affects normal implantation and decidualization in mice.

### 2.2. The Effect of DES on Preimplantation Embryo Development

**Figure 2:**
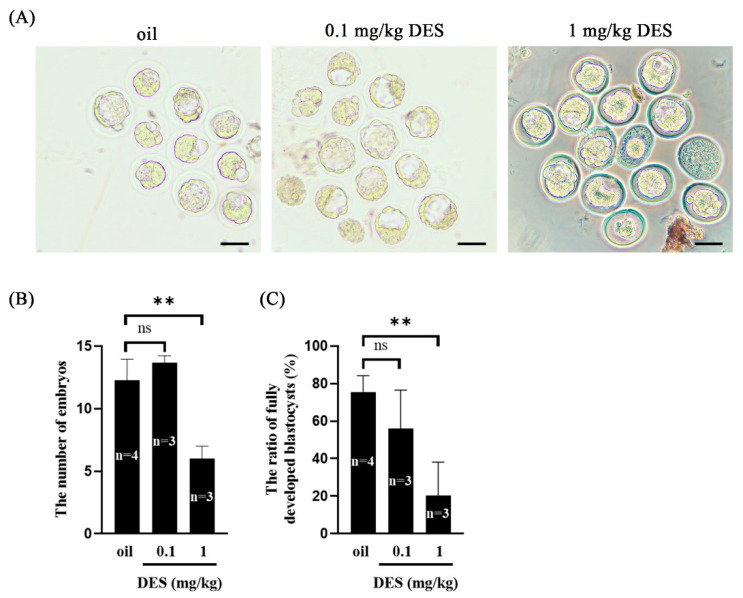
Effect of DES on preimplantation embryonic development in mice in vivo. (**A**) Morphology of embryos in control and DES-treated (0.1 mg/kg and 1 mg/kg) mice on day 4 of pregnancy. Scale bar = 100 μm. (**B**) The number of embryos collected from mouse uterus on day 4 of pregnancy. (**C**) The proportion of embryos developing into complete blastocysts per mouse on day 4 of pregnancy. *n*, the number of mice used. **, *p* < 0.01; ns, not significant.

Because DES has adverse effects on mouse implantation and decidualization, we wanted to know whether DES affects mouse embryonic development. Embryos were flushed out from the uterus of mice on day 4 of pregnancy. Compared with the control group and the 0.1 mg/kg DES group, the number of embryos washed out on day 4 of pregnancy in the 1 mg/kg DES group was reduced (Figure 2B). Interestingly, the proportion of fully developed blastocysts per mouse in the 1 mg/kg DES group was significantly reduced (Figure 2C). These results indicate that DES has adverse effects on preimplantation embryo development.

### 2.3. DES Causes Uterine Epithelial Abnormalities

**Figure 3:**
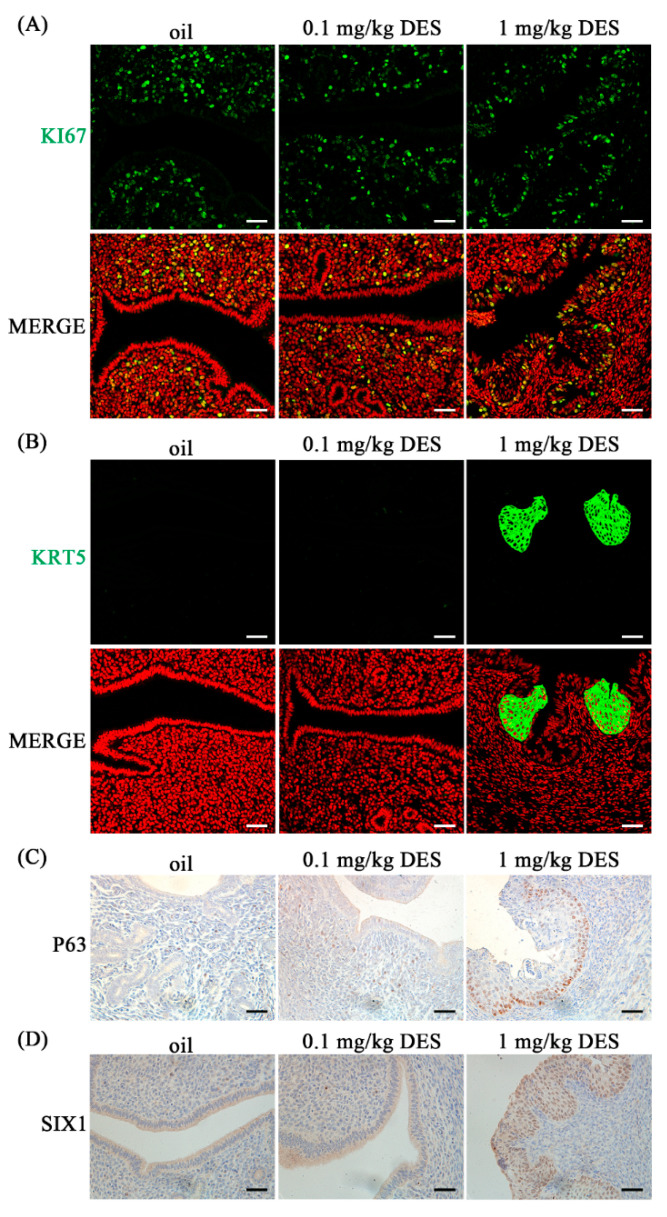
Immunofluorescence and immunostaining of uterine proliferation molecules in control and treatment groups (0.1 mg/kg DES and 1 mg/kg DES) on day 4 of pregnancy. (**A**) KI67 immunofluorescence. (**B**) KRT5 immunofluorescence. (**C**) P63 immunohistochemistry. (**D**) SIX1 immunohistochemistry. *n* = 3 mice. Scale bar = 50 μm.

On day 4 of pregnancy, preimplantation estrogen further enhances stromal cell proliferation, but epithelial cell proliferation ceased on day 4 [37]. KI67 is a recognized marker of cell proliferation [38]. Compared with the control group and 0.1 mg/kg DES group, KI67 immunofluorescence in uterine epithelial cells of the 1 mg/kg DES group was enhanced (Figure 3A). In contrast, KI67 signals in the stromal cells were weakened (Figure 3A). Cytokeartin 5 (KRT5) and P63 are biomarkers of stratified epithelial basal cells [39,40]. Compared with the control and 0.1 mg/kg DES group, the immunostaining signals of KRT5 and P63 in uterine epithelial cells of the 1 mg/kg DES group were increased (Figure 3B,C). Sine oculis homeobox homolog 1 (SIX1) is a potential biomarker of stratified epithelial basal cells and endometrial carcinogenesis [41,42]. Compared with the control and 0.1 mg/kg DES group, SIX1 immunostaining in uterine epithelial cells in the 1 mg/kg DES group was also enhanced (Figure 3D). These results suggest that DES may cause abnormal uterine epithelium proliferation and lesions.

### 2.4. DES Causes Abnormal Endometrial Receptivity

**Figure 4:**
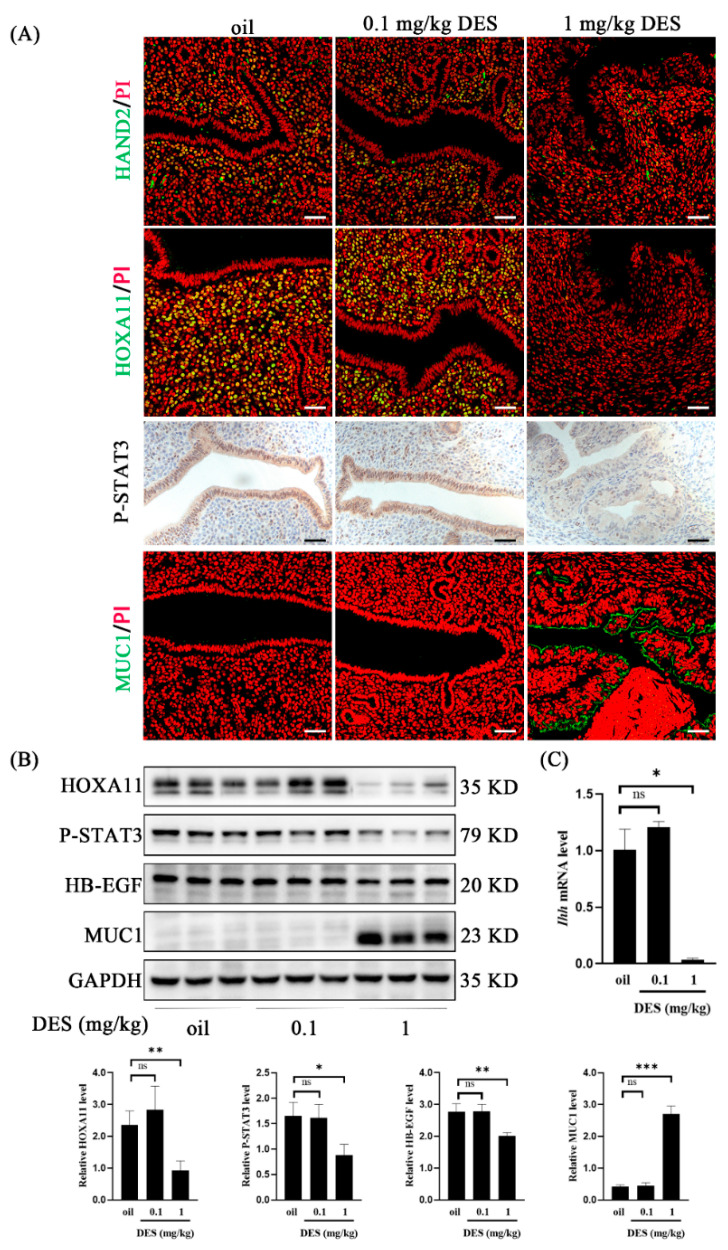
Analysis on implantation-related molecules in control group and DES-treated groups (0.1 mg/kg and 1 mg/kg) on day 4 of pregnancy. (**A**) HAND2, HOXA11, MUC1 immunofluorescence, P-STAT3 immunohistochemistry. Scale bar = 50 μm. (**B**) Western blot analysis of P-STAT3, HOXA11, MUC1, and HB-EGF protein levels. (**C**) QPCR analysis of Indian hedgehog gene *Ihh* mRNA level. *n* = 3 mice. *, *p* < 0.05; **, *p* < 0.01; ***, *p* < 0.001; ns, not significant.

Successful pregnancy requires the interaction between the activated embryo and the receptive uterus [43]. HAND2, Indian hedgehog (*Ihh*), signal transducer and activator of transcription 3 (STAT3) phosphorylation, homeobox A11 (HOXA11), and heparin-binding epidermal growth factor (HB-EGF) are recognized markers of uterine receptivity in mice [44,45,46,47]. In this study, HAND2, HOXA11, and P-STAT3 immunostaining were down-regulated in the 1 mg/kg DES group compared with the control group and the 0.1 mg/kg DES group (Figure 4A). HOXA11, P-STAT3, HB-EGF protein levels and the *Ihh* mRNA level were significantly decreased in the 1 mg/kg DES group (Figure 4B,C). The anti-adhesion molecule Mucin 1 (MUC1) is a recognized marker of uterine nonreceptivity in mice [48]. Compared with the control group and 0.1 mg/kg DES group, MUC1 immunofluorescence intensity and protein levels in the 1 mg/kg DES group were significantly increased (Figure 4A,B). These results indicate that DES may interfere with the normal uterine receptivity in mice.

### 2.5. DES Leads to the Activation of the Pyroptosis Pathway

**Figure 5:**
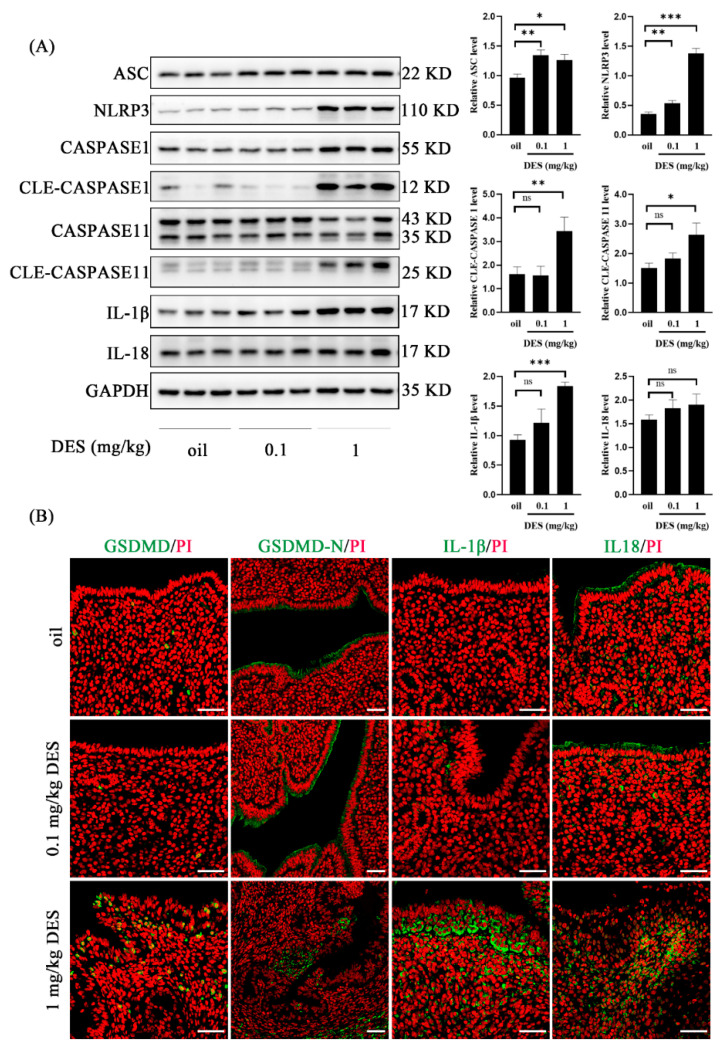
Changes in pyroptosis-related molecules in mouse uterus in the control and DES groups (0.1 mg/kg and 1 mg/kg) on day 4 of pregnancy. (**A**) Western blot analysis of ASC, NLRP3, CASPASE 1, cleaved CASPASE 1, CASPASE 11, cleaved CASPASE 11, IL-1β and IL-18 protein levels. (**B**) GSDMD, GSDMD-N, IL-1β, and IL-18 immunofluorescence. *n* =  3 mice. Scale bar = 50 μm. *, *p* < 0.05; **, *p* < 0.01; ***, *p* < 0.001; ns, not significant.

Appropriate pyroptosis is the key to embryo implantation and decidualization, while overactivated pyroptosis may lead to pregnancy failure [49]. To further explore the causes of DES-induced pregnancy loss, we detected the expression of pyroptosis-related molecules in mouse uterus on day 4 of pregnancy. Compared with the control group and 0.1 mg/kg DES group, the 1 mg/kg DES group significantly up-regulated the protein levels of ASC, NLRP3, cleaved CASPASE 1, cleaved CASPASE 11, and IL-1β (Figure 5A). However, the protein level of IL-18 did not change significantly in the 1 mg/kg DES group (Figure 5A). The immunofluorescence intensity of GDSMD, GSDMD-N, IL-1β, and IL-18 in the 1 mg/kg DES group was enhanced (Figure 5B). These results indicated that 1 mg/kg DES activated the classical inflammasome pathway NLRP3/CASPASE 1/GSDMD and the non-classical inflammasome pathway CASPASE 11/GSDMD.

## 3. Discussion

In our in vivo study in mice, the selection of 0.1 mg/kg and 1 mg/kg DES was mainly based on previous studies, in which 0.2 mg/kg DES increased the incidence of uterine tumors [50]. In vitro, we treated mouse stromal cells with 0.6 and 6 μM of DES. Mouse oocytes were treated with 5 μM, 20 μM, 40 μM, and 60 μM to interfere with their maturation [4,11]. Exposure to DES at 3–5 weeks of age reduced the number of corpus luteum and implantation sites in mice [51]. When rats were exposed to 45 μg/kg DES on days 18 to 20 of pregnancy, their offspring lost weight [52]. In our study, the 1 mg/kg DES group reduced the implantation sites on days 5 and 8 of pregnancy. Although 0.1 mg/kg DES had no significant effect on the implantation site on days 5 and 8 of pregnancy, it reduced the weight of implantation site on day 8 of pregnancy and the birth weight of offspring mice. Exposure to 50 μg/kg DES in mice resulted in decreased fertility of the F3 generation [53]. This indicates that DES may have a trans-generational effect. When mice were exposed to 1, 10, or 100 mg/kg DES from day 4 to day 8 of pregnancy, their decidual development was insufficient [54]. Our results showed that 6 μM DES inhibited decidualization in vitro. The early development of embryos directly determines the success or failure of the entire pregnancy [21]. *Zebrafish* embryos showed specific toxic effects after exposure to 0.1 μM DES [55]. In this study, 1 mg/kg DES reduced the number of embryos and delayed embryonic development in mice.

In our study, the uterine epithelium of mice exposed to 1 mg/kg DES showed squamous epithelium on day 4 of pregnancy, which may further develop into endometrial cancer. The main function of columnar epithelial cells is absorption and secretion, while stratified squamous epithelial cells form a barrier [56]. KRT5 and P63 were mainly expressed in squamous epithelial cells [57,58]. In this study, KRT5 and P63 were highly expressed in the uterine epithelium of mice in the 1 mg/kg DES group. Previous studies have shown that KRT5 is abnormally expressed in the prostate ducts of the offspring of DES-exposed mice [59]. P63 is also necessary for DES exposure-induced uterine epithelial squamousization [56]. SIX1 is usually present in the cervical and vaginal stratified squamous epithelium and is not expressed in the uterus [42]. When neonatal mice were exposed to DES, SIX1 is increased dramatically over time in the uteri at 6, 12, and 18 months of age and was associated with the development of endometrial cancer [41]. In this study, SIX1 was highly expressed in the uterine epithelium of mice in the 1 mg/kg DES group. Previous studies have also shown that SIX1 is present in endometrial luminal and glandular epithelial cells after neonatal exposure to DES [60]. DES exposure is associated with the incidence of breast cancer and cervical lesions in women [61]. When transgenic MT-mER mice overexpressing the estrogen receptor were exposed to 2 μg/g DES, the incidence of uterine adenocarcinoma was significantly higher than that of wild-type mice at 8 months of age [62]. When hamsters were exposed to DES on the day of birth, the incidence of uterine hyperplasia or dysplasia in adulthood reached 100%, and most of them developed into endometrial adenocarcinoma [63]. Our data suggest that DES exposure may lead to the transformation of uterine epithelium to squamous epithelium and may further develop into endometrial cancer.

Although the uterine receptivity of the 0.1 mg/kg DES group was normal, the uterine receptivity of the 1 mg/kg DES group was abnormal. Stat3 can be phosphorylated and translocated by Lif in the luminal epithelium during embryo implantation and Stat3 deficiency impairs embryo implantation [64]. The level of P-STAT3 was significantly down-regulated in the uterus of mice exposed to 1 mg/kg DES. Previous studies have shown that the P-STAT3 level is down-regulated in both Polycystic ovary syndrome (PCOS) mice and aging mouse models [44,65]. DES-induced uterine malformation may be due to the down-regulation of the HOXA11 gene [66]. The expression of HOXA11 was significantly down-regulated in the uterus of mice exposed to 1 mg/kg DES. Studies have found that the DES-induced mouse endometriosis model leads to the loss of HOXA11 expression [67]. Conventional knockout and uterus-specific deletion of HB-EGF in mice showed delayed embryo implantation and reduced litter size [68]. The expression of HB-EGF was down-regulated in the uterus of mice exposed to 1 mg/kg DES. Our data suggest that the failure of implantation in DES mice may be due to the disturbed expression of receptive molecules in the uterus.

The early pregnancy of mice exposed to 1 mg/kg DES may be affected by activating the classical pathway NLRP3/CASPASE 1/GSDMD and the non-classical pathway CASPASE 11/GSDMD. Pyroptosis is an inflammatory programmed cell death pathway [69]. Appropriate pyroptosis and inflammation are important for embryo implantation and pregnancy maintenance [70]. The ‘scratch method’ of endometrial injury before embryo transfer can induce local inflammation to help implantation [22]. However, excessive inflammatory response can lead to a variety of pregnancy complications, such as preeclampsia and abortion [71]. Adenosine triphosphate (ATP) is released at sites of tissue damage and inflammation [72]. ATP at high concentrations will inhibit decidualization in vitro [73]. LPS is a Gram-negative bacterial lipopolysaccharide that can cause inflammation. It can induce DNA damage in pre-implantation embryos and uterine cells, and ultimately may inhibit the implantation process in mice [74]. LPS causes excessive inflammation and leads to implantation failure by up-regulating the level of pyroptosis-related proteins [49]. In our study, pyroptosis-related proteins GSDMD-N, cleaved CASPASE 1, cleaved CASPASE 11, and IL-1β were significantly up-regulated in the uterus of 1 mg/kg DES mice. In addition, previous studies have shown that polystyrene microplastics cause pyroptosis in rat ovarian granulosa cells by up-regulating the NLRP3/CASPASE 1/GSDMD signaling pathway [75]. The endocrine disruptor BaP/BPDE induces pyroptosis and abortion in human trophoblast cells [76]. We infer that DES exposure may cause excessive inflammation, which leads to pregnancy failure.

## 4. Materials and Methods

### 4.1. Experimental Animals and Treatment Methods

ICR mice (6–8 weeks old) were purchased from Hunan Jingda Laboratory Animal Co., LTD. The mice were kept under temperature control conditions of 22 °C with the light cycle (12 h light and 12 h darkness). All mouse experiments were approved by the Animal Use and Care Committee of Guizhou University (EAE-GZU-2023-T005) on 3 March 2023.

Neonatal female pups were weighed daily and injected subcutaneously with 10 µL/g body weight (BW) of DES in sesame oil so that their final dose was 0.1 mg DES/kg BW (46207, Sigma-Aldrich, St. Louis, MO, USA) or 1 mg DES/kg BW or an equivalent volume of sesame oil as the control alone from postnatal day (PND) 1 to PND 5. After the mice reached sexual maturity (6 weeks), the female mice in each group mated with male mice of 8–12 weeks of age to induce pregnancy (the presence of vaginal plugs was day 1 of pregnancy). Uteri were collected at 10:00 a.m. on days 4, 5, and 8 of pregnancy, respectively. Half of the uterus was fixed in 10% formalin fixation solution, and the other half was frozen at −80 °C for further analysis. The birth weight and number of each litter in each group were recorded during parturition. On day 5 of pregnancy, 0.1 mL of 1% Chicago blue dye (Sigma-Aldrich, St. Louis, MO, USA) dissolved in normal saline was injected through the tail vein to determine the implantation site. On day 8 of pregnancy, the implantation site was weighed.

### 4.2. Immunofluorescence

Immunofluorescence was performed as previously described [44]. Mouse uterus was fixed in 10% neutrally buffered formalin and paraffin-embedded. The paraffin sections were dewaxed and rehydrated. The antigen retrieval was performed with EDTA buffer or citric acid solution by microwaving for 10 min. After cooling at room temperature, the sections were blocked with 10% horse serum at 37 °C for 1 h. Each primary antibody was incubated overnight at 4 °C. The primary antibodies used in this study include anti-MUC1 (1:100, Santa Cruz, Dallas, TX, USA), anti-HOXA11 (1:200, PA5-79387, Thermo Fisher Scientific, Waltham, MA, USA), anti-KI67 (1:200, ac230626086, Servicebio, Wuhan, China), anti-Cytokeartin 5 (1:200, ab52635, Abcam, Cambridge, UK), anti-HAND2 (1:100, sc-9409, Santa Cruz), anti-GSDMD (1:200, 334585, Abcam), anti-GSDMD-N (1:200, ER1901-37, HUABIO, Hangzhou, China), anti-IL-1β (1:200, ab254360, Abcam), and anti-IL-18 (1:200, ab207323, Abcam). After washing with PBS for three times, the sections were incubated with 488-conjugated secondary antibodies (2.5 mg/mL, G21234, Invitrogen, Carlsbad, CA, USA) for 30 min and counter-stained with propidium iodide (5 mg/mL, PI, P4170, Sigma-Aldrich). Fluorescence signals were collected using a Nikon laser scanning confocal microscope.

### 4.3. Immunohistochemistry

Immunohistochemistry was performed as previously described [44]. The mouse uterus was fixed in 10% neutrally buffered formalin and paraffin-embedded. The paraffin sections were dewaxed, rehydrated, and antigens were retrieved in Tris/EDTA buffer for 10 min. After cooling at room temperature, 3% hydrogen peroxide solution (85% methanol) was used to inhibit endogenous horseradish peroxidase (HRP) activity by 15 min. After washing with PBS 3 times, the sections were blocked in 10% horse serum at 37 °C for 1 h to prevent non-specific binding, and each antibody was incubated overnight at 4 °C. The primary antibodies used in this study included anti-P63 (1:50, ab124762, Abcam), anti-SIX1 (1:100, 10709-1-AP, Proteintech, Wuhan, China), and anti-P-STAT3 (1:100, 9131S, Cell Signaling Technology). After three washes with PBS, sections were incubated with matched biotin-labeled secondary antibody (1:200, Zhongshan Jinqiao, Beijing, China) and horseradish peroxidase-labeled streptavidin (1:200, Zhongshan Jinqiao) at 37 °C for 30 min, respectively. The positive signals were observed with a DAB horseradish peroxidase chromogenic kit (Zhongshan Jinqiao). The nuclei were counter-stained with hematoxylin.

### 4.4. Isolation and Treatment of Mouse Stromal Cells

Mouse endometrial stromal cells were isolated as previously described [49]. On day 4 of pseudopregnancy, mouse uterus was cut longitudinally, washed three times with HBSS, and placed in digestive solution (0.2% trypsin, 6 mg/mL dispase, 4.3 mL HBSS and 50 μL streptomycin/penicillin) at 4 °C for 1.5 h, room temperature for 30 min, and 37 °C for 10 min. The uterus was rinsed three times in HBSS, and the epithelium was rinsed off. The uterus was placed in 6 mL HBSS with 0.5% collagenase I (Invitrogen, 17100-017, Waltham, MA, USA) at 37 °C for 35 min. Endometrial stromal cells were collected and inoculated on a culture plate containing DMEM/F12 containing 10% fetal bovine serum. Stromal cells were treated with estradiol-17β (10 nM) and progesterone (1 μM) in DMEM/F12 containing 2% charcoal-treated FBS (cFBS, Biological Industries, Cromwell, CT, USA) to induce decidualization in vitro. Under in vitro decidualization, stromal cells were treated with DES (0.6 μM, 6 μM) for 24 h, 48 h, and 72 h, respectively.

### 4.5. Western Blot

Western blot was performed as previously described [49]. The protein concentrations were quantified by the BCA method (Thermo Fisher Scientific, Waltham, MA, USA). Protein samples (10 μg) were run on 12% SDS-polyacrylamide gel electrophoresis (SDS/PAGE) and transferred to PVDF membrane (IPVH00010, Millerica, MA, USA). The PVDF membrane was blocked with 5% skim milk at room temperature for 1 h, and incubated with each primary antibody in a shaking bed at 4 °C overnight. The antibodies used in this study included anti-GAPDH (1:1000, SC-32233, Santa Cruz), anti-P-STAT3 (1:1000, 9131s, Cell Signaling Technology, Danvers, MA, USA), anti-HB-EGF (1:1000, RQ7309, NSJ bio, Minnetonka, Minnesota, USA), anti-MUC1 (1:1000, ab45167, Abcam), anti-HOXA11 (1:1000, NBP1-80228, NOVUS, St. Louis, Missouri, USA), anti-ASC (1:1000, ab309497, Abcam), anti-NLRP3 (1:1000, NBP2-12446, NOVUS), anti-CASPASE 1 (1:1000, ab179515, Abcam), anti-CASPASE 11 (1:1000, ab180673, Abcam), anti-IL-1β (1:1000, ab254360, Abcam), and anti-IL-18 (1:1000, ab207323, Abcam). The membrane was incubated with a secondary antibody (1:5000, Invitrogen) coupled with horseradish peroxidase (HRP) at room temperature for 1 h, and the signals were developed with an ECL chemiluminescence kit (Millipore, Saint Louis, MO, USA) using the 5200 Tanon imaging system. The Western blot signal strength was detected by ImageJ software (Image J 1.52). The level of each band for Western blot was normalized to the level of GAPDH.

### 4.6. Real-Time RT-PCR

Real-time PCR was performed as previously described [77]. The total RNAs of tissues or cells were extracted using the Trizol reagent kit (9109, Takara, Kusatsu, Japan), and genomic DNA was digested with RQ1 deoxyribonuclease I (Promega, Fitchburg, WI, USA) to purify RNA. The PrimeScript Reverse Transcriptase Kit (R222-01-AB, Vazyme, Nanjing, China) was used to reverse transcribe 0.5 μg of RNA into cDNA from each sample, as per the manufacturer’s instructions. For real-time PCR, cDNA (1.2 μg) was amplified using a SYBR pre-mixed Ex Taq kit (Q311-02-AA, Vazyme). The data were analyzed by the 2^−ΔΔCt^ method and normalized to the level of *Rpl7* in mice. The primer sequences mentioned in this work are as follows: *Rpl7* (sense: GCAGATGTACCGCACTGAGATTC and antisense ACCTTTGGGCTTACTCCATTGATA); *Prl8a2* (sense: AGCCAGAAATCACTGCCACT and antisense TGATCCATGCACCCATAAAA); *Ihh* (sense: GCTGAAGGGACTCTAACC and antisense ACAGAGGACGGAGACAAC).

### 4.7. Statistical Analysis

Data were statistically analyzed using GraphPad Prism 8 software. The Student’s *t* test was employed to compare the difference between the two groups. Each group in the mouse study contained a minimum of three mice. Data were expressed as the mean ± standard deviation (SD). The two groups were compared by the Student’s *t* test. The one-way ANOVA test was used to compare multiple groups. Statistical significance was defined as *, *p* < 0.05; **, *p* < 0.01; ***, *p* < 0.001; ns, not significant.

## 5. Conclusions

Our study shows that exposure to DES is not conducive to the establishment of early pregnancy in mice, especially uterine receptivity and decidualization. DES leads to the abnormal activation of the pyroptosis pathway.

## Data Availability

Data supporting the findings of this study are available within the paper.
